# Manipulation of epsilon-near-zero wavelength for the optimization of linear and nonlinear absorption by supercritical fluid

**DOI:** 10.1038/s41598-021-95513-6

**Published:** 2021-08-05

**Authors:** Jiaye Wu, Xuanyi Liu, Haishi Fu, Kuan-Chang Chang, Shengdong Zhang, H. Y. Fu, Qian Li

**Affiliations:** 1 School of Electronic and Computer Engineering, Peking University, Shenzhen, 518055 China; 2 Tsinghua Shenzhen International Graduate School, Tsinghua University, Shenzhen, 518055 China

**Keywords:** Optical materials and structures, Optical techniques, Optics and photonics, Optical physics, Nanophotonics and plasmonics, Nonlinear optics, Sub-wavelength optics

## Abstract

We introduce supercritical fluid (SCF) technology to epsilon-near-zero (ENZ) photonics for the first time and experimentally demonstrate the manipulation of the ENZ wavelength for the enhancement of linear and nonlinear optical absorption in ENZ indium tin oxide (ITO) nanolayer. Inspired by the SCF’s applications in repairing defects, reconnecting bonds, introducing dopants, and boosting the performance of microelectronic devices, here, this technique is used to exploit the influence of the electronic properties on optical characteristics. By reducing oxygen vacancies and electron scattering in the SCF oxidation process, the ENZ wavelength is shifted by 23.25 nm, the intrinsic loss is reduced by 20%, and the saturable absorption modulation depth is enhanced by > 30%. The proposed technique offers a time-saving low-temperature technique to optimize the linear and nonlinear absorption performance of plasmonics-based ENZ nanophotonic devices.

## Introduction

Epsilon-near-zero (ENZ) materials have received vast scientific attentions^1–8^ in the past decade, whose permittivity can reach a near-zero value, enabling extraordinary optical properties such as phase tunneling^9^ and pattern tailoring^10^, field^11^ and nonlinearity^12^ enhancement, slow-light effect^13^, pulse shaping^14^, quasi-standing-wave pattern in subwavelength self- and pulse-matter interactions^15,16^, strong absorption^17,18^, impact of losses^19^, frequency conversion^20,21^, etc. Subsequently, these features give rise to various photonic applications, for instance, on-chip quantum networks^22^, nanoantennas^23,24^, all-optical switching^25–28^, electro-, and all-optical modulators^29–32^, etc.

With appropriate free carrier concentration, transparent conducting oxides (TCOs) operating at the bulk plasmon frequency allow ENZ to be realized in different spectral regions, from ultraviolet to infrared^1,3^. Among various kinds of TCOs, indium tin oxide (ITO) is frequently used due to its low-cost, high stability, and commercial availability. It is also well acknowledged for its high Kerr nonlinearity^33^, second-^19,34,35^, third-^19,35,36^, high-order^37–40^ harmonic, and terahertz^41^ generations. Besides these demonstrations which focus more on the ultrafast and light-matter interactions of the ENZ materials and the probe/pump light themselves, means to borrow and apply techniques that influence, control, assist, and boost the interactions are equally important and interesting to study. This kind of investigations can deepen people’s understanding of the underlying physical mechanisms, and provide opportunities for novel ideas and new optical phenomena.

Most of these exotic linear and nonlinear optical properties exist in the proximity of the ENZ wavelength ($\lambda _{\mathrm{ENZ}}$) where the real part of the complex permittivity reaches zero, which requires the alignment of the sample’s $\lambda _{\mathrm{ENZ}}$ with the light source. By applying a voltage bias^29^, the free carrier concentration can be temporarily tuned to change $\lambda _{\mathrm{ENZ}}$, but this technique requires external voltage support, limiting its application in all-optical setups. Another more permanent approach is to strictly control the conditions in the fabrication processes. In these processes, factors like the atmosphere, deposition rate, oxygen flow speed, temperature and time of annealing, etc., can all influence the actual free carrier concentration and mobility in many different ways, which will in turn, affect the optical properties of the ITO film in the ENZ region. Recently, useful controlled annealing methods are demonstrated^42,43^ to tune $\lambda _{\mathrm{ENZ}}$ of the TCOs over a relatively wide spectral range. These techniques require a relatively high temperature (e.g. 350 °C) and long processing time (e.g. 2 hours), and they do not deliberately optimize either the linear and nonlinear optical loss. Additionally, TCOs are known to exhibit *high intrinsic loss* in the vicinity of $\lambda _{\mathrm{ENZ}}$, which is hard to reduce and poses a great challenge in their applications in large-scale integrated photonic platforms^44^. Therefore, adjusting the ENZ region and its intrinsic loss to a certain value to fulfil a specific demand or application can be very time-consuming and inconvenient.

The state-of-the-art supercritical fluid (SCF) technology is widely applied in food science, environmental science, natural product extraction, pharmaceutics^45,46^, and especially, micro- and nano-electronic devices^47–52^. By applying proper pressure and temperature, carbon dioxide (${\text {CO}}_2$) can easily enter the phase of SCF^53^, where it can permeate through solid-state structures to repair lattice defects, reconnect chemical bonds, adjusting carrier mobility, and introduce dopants. The SCF treatment has been proved effective in improving the performance of microelectronic semiconductor devices with ITO electrodes^51,52^, and this very technique might also shed light on the manipulation of its optical ENZ properties, which are also dopant- and carrier-mobility-tuning-related. We believe that it would be interesting and helpful to introduce this technique to optics and ENZ photonics, and explore the possibility of the post-fabrication optimization of the ENZ properties in ITO nanolayers.

In our seminar work^54^, we preliminarily demonstrated that SCF extraction can have an impact on the linear optical properties of ENZ TCOs. Here, we further present a more in-depth investigation and analyses on the SCF oxidation’s manipulation of $\lambda _{\mathrm{ENZ}}$ for the optimization of the linear and nonlinear optical absorption characteristics of ENZ ITO nanolayer, as well as its underlying physical mechanisms. A competing mechanism theorem is proposed and analyzed to hypothesize different processes involved, which might cause contradicting outcomes in the directions of $\lambda _{\mathrm{ENZ}}$ shifting and the variation of loss. It is demonstrated that by the stable, time-saving (1 hour), and low-temperature ($120^\circ \hbox {C}$) SCF oxidation technique, $\lambda _{\mathrm{ENZ}}$ of the ITO nanolayer is stably shifted by 23.25 nm, the intrinsic loss in ENZ region is reduced by 20%, and the modulation depth (MD) of the saturable absorption (SA) is enhanced by > 30%. This work also demonstrates the *first-time direct observation* of the influences of multiple electrical parameters on the linear and nonlinear properties via Drude model, which could bring deeper understandings and insights into optical physics, ENZ photonics, TCOs, and further explorations in ENZ SCF techniques.

**Figure 1:**
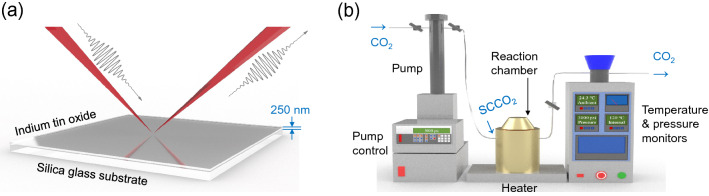
Schematic diagrams of the sample and the SCF processing. (**a**) The ITO nanolayer sample under VASE measurement; (**b**) The SCF processing system with pressure and temperature control and monitoring.

## Results and discussions

### Theory and hypothesis

In theory, for ITO nanolayer, its complex permittivity can be well-described by the Drude model^55^ in the ENZ region:

1 $$\begin{aligned} {\varepsilon _{\mathrm{R}}}\left( \omega \right) = {\varepsilon _{\mathrm{r}}} + i{\varepsilon _{\mathrm{i}}} = {\varepsilon _{\mathrm{b}}} - \frac{{\omega _{\mathrm{p}}^2}}{{\left( {{\omega ^2} + {\gamma ^2}} \right) }} + i\frac{{\omega _{\mathrm{p}}^2\gamma }}{{\left( {{\omega ^2} + {\gamma ^2}} \right) \omega }}, \end{aligned}$$

where $\varepsilon _{\mathrm{r}}$ and $\varepsilon _{\mathrm{i}}$ are the real and imaginary part of the permittivity, and the imaginary part denotes the intrinsic loss of the material. $\varepsilon _{\mathrm{b}}$ is the background (high frequency) permittivity, $\omega$ is the angular frequency of the electromagnetic wave, $\omega _{\mathrm{p}}$ is the plasma frequency, and $\gamma$ is the Drude relaxation rate which represents the damping rate of the free electrons related to the scattering loss and scales directly with $\varepsilon _{\mathrm{i}}$. $\omega _{\mathrm{p}}$ and $\gamma$ are determined by the following relations.

2 $$\begin{aligned} \begin{array}{l} \displaystyle {\omega _{\mathrm{p}}} = \sqrt{\frac{{N{e^2}}}{{{\varepsilon _0}{m^*}}}} , \\ \displaystyle \gamma = \frac{e}{{\mu {m^*}}}. \end{array} \end{aligned}$$

Here, *N* is the free carrier concentration, $e = 1.6\times 10^{-19} \, \hbox {C}$ is the amount of charge for an electron, $\varepsilon _0 = 8.85\times 10^{-12} \, \hbox {F/m}$ is the vacuum permittivity, $m^*$ is the effective mass of an electron, and $\mu$ is the mobility of free carrier.

As a popular material for realizing ENZ, ITO often exhibits a spectral ENZ point ($\lambda _{\mathrm{ENZ}}$) where $\varepsilon _{\mathrm{r}}$ reaches zero at NIR and a non-zero $\varepsilon _{\mathrm{i}}$ which is not small. Tuning the wavelength of $\lambda _{\mathrm{ENZ}}$ to a specific value requires multiple attempts to find the optimal deposition conditions. This can be very time consuming and inconvenient, and by this technique the fine-tune control ($< 100 \, \text {nm}$) of $\lambda _{\mathrm{ENZ}}$ is nearly impossible. On the other hand, the non-zero large absorption also limits its applications in integrated photonics, which is a difficulty yet to tackle in the field of optics. Therefore, in this work we focus on the manipulation and enhancement of the linear and nonlinear optical ENZ properties in its ENZ regime.

**Figure 2:**
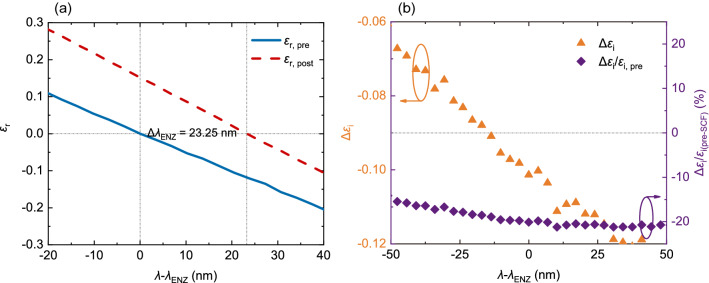
Linear ENZ properties manipulated and improved by SCF. (**a**) The change in $\lambda _{\mathrm{ENZ}}$ after SCF oxidation process. The solid line represents the real part of permittivity $\varepsilon _{\mathrm{r}}$ before the SCF processing, and the dashed line denotes that after SCF. (**b**) The change in loss within the ENZ region. The triangles represent the changes of imaginary parts $\Delta \varepsilon _{\mathrm{i}} = \varepsilon _{\mathrm{i, post}} - \varepsilon _{\mathrm{i, pre}}$ (refer to the left *y*-axis), and the diamonds denote the proportions of changes compared with the $\varepsilon _{\mathrm{i}}$ values before SCF (refer to the right *y*-axis).

In light of its ability to affect dopants, chemical bonds, and carrier mobility, we consider SCF treatment a promising choice to manipulate the ENZ properties of ITO. Here we present SCF oxidation technique (see “Methods” section). The hypothesis is: SCF oxidation will reduce the oxygen vacancies (which indicates excessive metal ions and dangling chemical bonds) in ITO and thus reduce the free carrier concentration *N*. By repairing and re-connecting broken bonds, the scattering of electrons can be reduced, leading to a better mobility $\mu$. For the real permittivity, according to Eq. (2) this will decrease $\omega _{\mathrm{p}}$ and lead to an increase in $\varepsilon _{\mathrm{r}}$ (corresponding to the red-shift of $\lambda _{\mathrm{ENZ}}$) in Eq. (1). For the loss part, in Eq. (1), the loss $\varepsilon _{\mathrm{i}}$ is predicted to decrease with $\omega _{\mathrm{p}}$, and an improved $\mu$ can also contribute to a lower loss. However, another competing mechanism exists. Supercritical ${\text {CO}}_2$ (${\text {SCCO}}_2$) can carry the initial dopant, ${\text {H}}_2 \text {O}$ molecules, into the ITO nanolayer, which is known to provide extra free carriers, resulting in an increased *N* and a blue-shifted $\lambda _{\mathrm{ENZ}}$. ${\text {H}}_2 \text {O}$ has a strong absorption peak near 1200 nm, which lies in ITO’s ENZ regime and can cause a higher loss $\varepsilon _{\mathrm{i}}$. These two contradicting mechanisms compete under the given experimental conditions, and it is hard to identify the dominant one by the theory alone. This hypothesis is called the “competing mechanism theorem” of TCO SCF processing, and the dominant effect will determine the actual change in optical ENZ properties. This theorem can exist in TCOs for nearly all known SCF techniques, not limited to SCF oxidation.

### ENZ property manipulation by SCF oxidation

The 250-nm thick ITO nanolayers were deposited on pure silica glass by DC magnetron sputtering using 99.99% purity 10 wt% ITO target. The samples were then annealed in vacuum at 350 °C for 2 h to elevate free electron concentration so as to exhibit optical ENZ. The complex permittivity curves of the samples are measured by a variable angle spectroscopic ellipsometer (VASE) and $\lambda _{\mathrm{ENZ}}$ is observed at 1200 nm (see Supplementary Information). Figure 1a illustrates the fabricated ITO nanolayer sample under VASE measurement, and Fig. 1b shows the schematic diagram of the SCF setup. Pure ${\text {CO}}_2$ is pressurized to 3000 psi by the pump. Subsequently, the chamber is heated to 120 °C, and the compressed ${\text {CO}}_2$ is pumped into it, turning into ${\text {SCCO}}_2$, and starts the reaction. The temperature and pressure monitors ensure the experiments are kept under correct conditions. The processing lasts for 1 hour, and when the reaction is finished, ${\text {SCCO}}_2$ is depressurized via a two-level valve system. Before and after the SCF processing, the ITO nanolayer samples are measured by VASE, Fourier-transform infrared (FTIR) spectrometer, Hall effect, and a figure-9 fiber laser SA testing system (see “Methods” section). Scanning electron microscope (SEM) and X-ray photoelectron spectroscopy (XPS) are also used to aid the elemental analyses.

The results of SCF processing are shown in Fig. 2. In Fig. 2a, the solid line is the original $\varepsilon _{\mathrm{r}}$ curve and the dashed line denotes the one after SCF treatment. Due to the fact that the intrinsic loss highly depends on fabrication processes, for clarity and without loss of generality, the absolute difference of $\varepsilon _{\mathrm{i}}$ before and after SCF process, and the portion of improvement are plotted in Fig. 2b. The triangles denote the absolute differences of the measured $\varepsilon _{\mathrm{i}}$ data, and the diamonds represent the percentage of $\varepsilon _{\mathrm{i}}$ change in the 100-nm-wide ENZ region in the proximity of $\lambda _{\mathrm{ENZ}}$. The curves of $\varepsilon _{\mathrm{i}}$ values can be found in Fig. S1 in Supplementary Information. As shown in the figure, after the SCF oxidation processing, $\lambda _{\mathrm{ENZ}}$ of the sample red-shifts 23.25 nm, while its linear loss in the 100-nm proximity of $\lambda _{\mathrm{ENZ}}$ represented by the ratio between $\Delta \varepsilon _{\mathrm{i}}$ change and the pre-SCF value $\varepsilon _{\mathrm{i, pre}}$ reduced by 20%. These results are stable in our repeated experiments (see Supplementary Information) and they indicate that, in the hypothesis, the dominant effect here is the occupation of oxygen vacancies. The reduction of defects and free carrier concentration *N* lead to a reduction of $\omega _{\mathrm{p}}$ and an improvement of $\mu$, which translates to a red-shifted $\lambda _{\mathrm{ENZ}}$ and a declined loss. It is worth noting that the reduction of loss not only exists in the ENZ region, but extends from $\sim 1100 \, \hbox {nm}$ towards 1690 nm (the maximum wavelength that could be measured by VASE). This is particularly useful for TCO-based photonic applications that are limited by the huge optical loss. Additional Hall effect measurement show that the mobility $\mu$ of the sample increases from $9.36 \, {\hbox {cm}}^2/(\hbox {V} \cdot \hbox {s})$ to $12.25 \, {\hbox {cm}}^2/(\hbox {V} \cdot \hbox {s})$, and the free carrier concentration decreases from $1.834 \times 10^{21} \, {\hbox {cm}}^{-3}$ to $1.391 \times 10^{21} \, {\hbox {cm}}^{-3}$. By Eq. (2), it can be calculated that $\omega _{\mathrm{p}}$ drops from $3.9139 \times 10^{15} \, \hbox {rad/s}$ to $3.4085 \times 10^{15} \, \hbox {rad/s}$, while $\gamma$ declines from $4.9387 \times 10^{14} \, \hbox {rad/s}$ to $3.7731 \times 10^{14} \, \hbox {rad/s}$, which is consistent with the provided explanation and hypothesis. This partially confirms the analyses above, and the success in dopant introduction can be seen in the FTIR spectra and XPS results, which will be discussed in “Elemental analyses” section.

**Figure 3:**
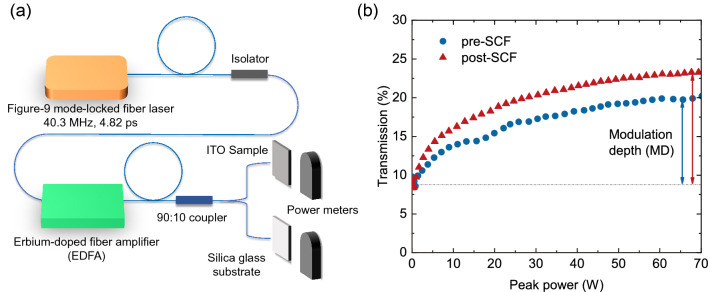
Enhancement of the SA characteristics. (**a**) Scheme of the fiber laser SA testing system. (**b**) Saturable absorption characteristics of the SCF-processed sample.

### Nonlinear saturable absorption properties

Besides SCF’s ability to manipulate the linear ENZ optical properties, it can also improve the samples’ nonlinear SA properties. Here we test the SA properties at 1550 nm, the telecommunication window, which belongs to the greater ENZ region ($\varepsilon _{\mathrm{r}}\approx -1$). A figure-9 40.3-MHz repetition rate 4.82-ps fiber laser adapted from our previous work^56^ is used to obtain the SA characteristics. The scheme of the laser system is shown in Fig. 3a with further information in the “Methods” section, and the SA results are shown in Fig. 3b where the circles denote the measured data before SCF processing, and the triangles are those after the treatment.

**Figure 4:**
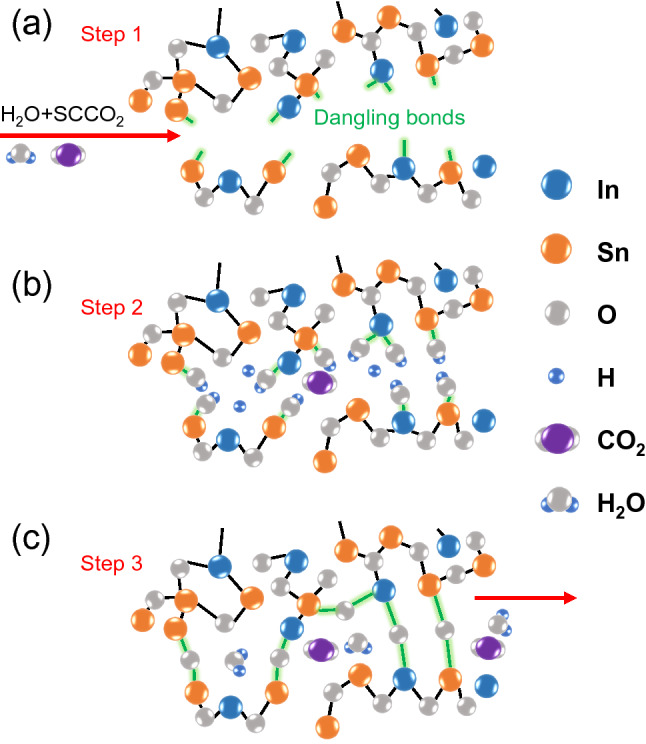
The SCF oxidation reaction processes. (**a**) The ${\text {H}}_2 \text {O}$ dissolved in ${\text {SCCO}}_2$; (**b**) The lattice-${\text {H}}_2 \text {O}$ interactions; (**c**) The dihydroxylation process and the occupation of oxygen vacancy.

**Figure 5:**
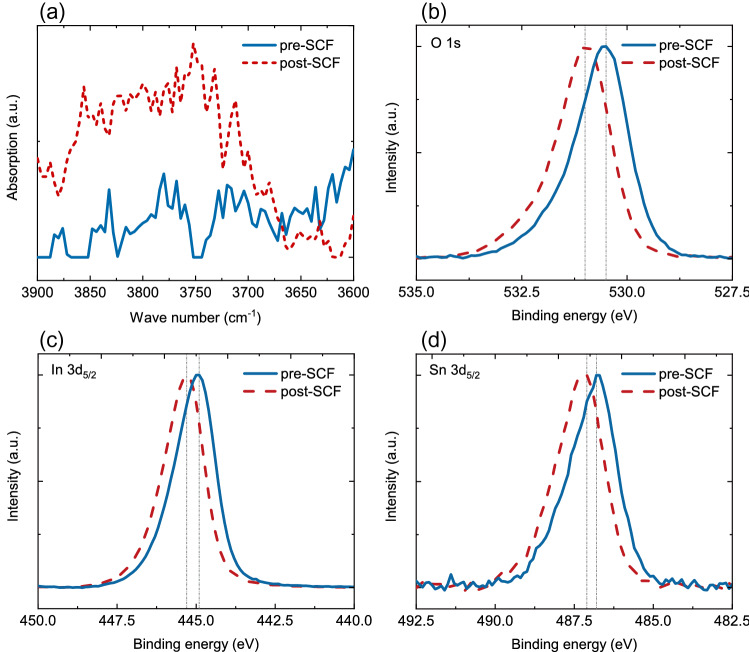
FTIR and XPS analyses. (**a**) The FTIR results before and after SCF processing. The XPS results of (**b**) O 1s, (**c**) In ${ {3d}}_{5/2}$, and (**d**) Sn ${ {3d}}_{5/2}$ before and after SCF processing.

The difference between the initial transmission and the saturated transmission is defined as the MD, which denotes the ON–OFF contrast ratio of a modulation or switching device, with the higher the MD, the better the performance. Before SCF oxidation, the transmission starts at 8.76% and saturates at 19.92%, yielding an MD of 11.16%. After processing, the saturated transmission is 23.28%, indicating an MD of 14.52% and an improvement of 30.11%. The SA phenomenon happens when the intensity of the incident light is sufficiently high that it excites atoms from the ground state to an upper energy state at an ultrafast rate. The rate is so fast such that the atoms have no time to fall back to the ground state; thus, the ground state is depleted when the nonlinear absorption saturates. Similar to the excitation of electrons from the valence band into the conduction band in semiconductors, when lattice defects exist, electrons can be trapped in a middle state, the defect-induced “deep state”, which reduces the number of electrons that can be excited to conduction band. Here in the SA phenomenon of ITO, oxygen vacancies and dangling chemical bonds form a metastable energy band between the upper stable state and the ground state. When those defects are reduced, the intermediate metastable energy band will split into two tailing bands which expand the density of states for the ground and upper state (See Supplementary Information). This helps improve the SA characteristics and results in a greater MD. The enhancement shown here is significant, and can be potentially beneficial for loss-operated nanophotonic devices like all-optical switches and modulators.

### Elemental analyses

The reaction processes of SCF oxidation can account for the linear and nonlinear optical property changes in the ENZ ITO nanolayer. Figure 4 is a schematic diagram of the reactions in SCF oxidation, which mainly consists of three steps: (i) the ${\text {SCCO}}_2$ dissolves ${\text {H}}_2 \text {O}$, and they permeate into the ITO structure (Fig. 4a); (ii) ${\text {H}}_2 \text {O}$ interacts with the dangling bonds and lattice defects (Fig. 4b); (iii) dihydroxylation process re-produces ${\text {H}}_2 \text {O}$ and leaves oxygen in ITO, and the oxidation is completed (Fig. 4c). Detailed reaction formula can be found in the “Methods” section.

FTIR and XPS results of the ENZ ITO nanolayer before and after SCF processing are shown in Fig. 5, where the solid lines show the pre-SCF data, and the dashed lines denote the post-SCF data. The FTIR results in Fig. 5a show typical oscillating absorption peaks of ${\text {H}}_2 \text {O}$ and O-H from $3600 \, {\hbox {cm}}^{-1}$ to $3900 \, {\hbox {cm}}^{-1}$ for the sample after SCF processing. Stronger evidence can be found in the XPS results. In Fig. 5b, the peak of binding energy of O 1*s* increases from 530.5 to 531 eV, exhibiting a decrease in its average chemical valence. This means that the amount of suspending non-lattice oxygen increases, which is the result of oxygen introduction by SCF. In the meantime, from Fig. 5c and d, In ${{3d}}_{5/2}$ and Sn ${{3d}}_{5/2}$ also see increases in their binding energy. The binding energy for In ${{3d}}_{5/2}$ rises to 445.3 eV from 444.9 eV, and that for Sn ${{3d}}_{5/2}$ reaches 487.1 eV from 486.8 eV. From the XPS analysis, the atomic ratio between the two major elements O:In also climbs from 1.5255 to 1.5357. These results indicate that the “degrees of oxidation” for In and Sn become higher after the SCF processing, which are another two strong proofs of a successful oxygen introduction. Additionally, SEM results (see Supplementary Information) show no significant change occurs on the surface profile of the ITO nanolayer, meaning that the SCF treatment is a damage-free method that can be safely implemented in future applications.

## Conclusions

In summary, we introduce the techniques of SCF into ENZ photonics for the first time as a time-saving, low-temperature, and higher precision manipulation method. We experimentally demonstrate the manipulation of the $\lambda _{\mathrm{ENZ}}$, the reduction of the linear loss, and the improvement of the SA modulation depth for the 250-nm thick ENZ ITO nanolayer. By analyzing and hypothesizing the distinctive competing mechanisms with opposite results, one can obtain a clear idea of the physical processes involved from the results of the experiments. The dominant one will dictate the shifting direction of $\lambda _{\mathrm{ENZ}}$ and the change of optical loss. By exploiting the defect-repairing and doping function of SCF treatment, it is demonstrated that the oxidation process can shift $\lambda _{\mathrm{ENZ}}$ of the ITO nanolayer by 23.25 nm, reduce the intrinsic loss in ENZ region by 20%, and increase the MD of its nonlinear SA characteristics at 1550 nm by 30.11%, which is very useful in boosting the performance of ENZ TCO nanophotonic devices. Conclusively, the proposed technique has a significant effect on the linear and nonlinear optical properties on ENZ ITO nanolayer, and similar behaviors are expected in other ENZ TCOs. This work also reveals a practical, time-saving, and low-temperature technique that could be used in plasmonic photonics and inspire further endeavors.

## Methods

### Permittivity measurement

A J.A. Woollam M-2000UI variable angle spectroscopic ellipsometer (VASE) is used to measure and transform the results into complex permittivity from 246.2–1689.2 nm. Each sample is measured 4 times independently to rule out the measurement errors. The mean squared error (MSE) between the data and fitting model in the ENZ region is around 22.286–34.041. The determination of $\lambda _{\mathrm{ENZ}}$ is explained in Supplementary Information. Bare silica glass substrates go through the same SCF treatment with the ITO samples. Measurements are performed on both the ITO and the glass before and after the SCF treatment. No changes are observed in the glass substrate that would affect the optical properties of ITO.

### Hall effect measurement

An HMS-3000 Hall effect measurement system by Ecopia is used to measure the mobility of the samples before and after SCF processing.

### Nonlinear SA characterization

The measurement on the SA characterization of the ITO nanolayers are performed using a figure-9 40.3-MHz repetition rate 4.82-ps fiber laser at 1550 nm. The laser system consists of a figure-9 laser cavity, an optical isolator, an EDFA, a 90:10 coupler, sample holders, and power meters. The output power of the system is adjusted by the turning the waveplate in the cavity and controlling the pump power in the EDFA.

### FTIR measurement

PerkinElmer FTIR spectrometer is used to determine whether there are changes of functional groups before and after SCF processing (dopant introduction). FTIR results on the substrate can be found in Supplementary Information.

### XPS characterization

An ESCALAB 250Xi XPS by Thermo Fisher is used to probe the changes in binding energy for oxygen, indium, and stannum (tin).

### SEM characterization

A ZEISS SUPRA 55 SEM from Carl Zeiss is used to observe the surface profile of the ENZ ITO nanolayer before and after SCF processing. The purpose is to see whether SCF can cause any surface damage that might compromise its optical performance in application. Detailed comparisons of the SEM photographs (pre- and post-SCF) are shown in Supplementary Information.

### SCF oxidation technique

${\text {SCCO}}_2$ as the solvent with $125 \, \mu \hbox {L} \, {\text {H}}_2 \text {O}$. The temperature is set to $120^\circ \hbox {C}$, and the pressure is 3000 psi ($\sim 20 \, \hbox {MPa}$), with 1 hour of reaction time. In the reaction chamber, the amount of the added ${\text {H}}_2 \text {O}$ is excessive, and the possible oxidation bonding reactions^47–52^ are: $2\hbox {Sn/In-O} + {\text {H}}_2 \text {O}$ (SC) $\rightarrow$ 2Sn/In-OH and $2\hbox {Sn/In-OH} \rightarrow \hbox {Sn/In-O-Sn/In} + {\text {H}}_2 \text {O}$(SC). The determination and selection of SCF reaction parameters is explained in Supplementary Information.

## Supplementary Information

Supplementary Information.

## Data Availability

The data that support the findings of this study are available from the corresponding author upon reasonable request.
