# Supplementary material for: Manipulation of epsilon-near-zero wavelength for the optimization of linear and nonlinear absorption by supercritical fluid
Source: Sci Rep. 2021 Aug 5;11:15936. doi: 10.1038/s41598-021-95513-6 (PMC8342460; doi:10.1038/s41598-021-95513-6)
Supplement: Supplementary file 1 — Supplementary Information. [file 41598_2021_95513_MOESM1_ESM.pdf]

Supplementary Information for:

Manipulation of epsilon-near-zero wavelength for the optimization of linear and nonlinear absorption by supercritical fluid

Jiaye Wu[1], Xuanyi Liu[2], Haishi Fu[1], Kuan-Chang Chang[1], Shengdong Zhang[1], H. Y. Fu[2], and Qian Li[1,*]

[1]School of Electronic and Computer Engineering, Peking University, Shenzhen 518055, China.
[2]Tsinghua Shenzhen International Graduate School, Tsinghua University, Shenzhen 518055, China.
[*]Corresponding author. Email: liqian@pkusz.edu.cn

This document is the supplementary information for *Manipulation of epsilon-near-zero wavelength for the optimization of linear and nonlinear absorption by supercritical fluid* on the determination of λ_{ENZ} and experimental parameters, SEM results, and FTIR results of substrate.

S1. Obtaining the λ_{ENZ} and the values of ε_i

S2. Determination of experimental parameters

S3. SEM results

S4. FTIR results of substrate

S5. Visualize the SCF's enhancement on the MD of nonlinear SA

Figure S1. Intrinsic loss in the ENZ region before and after SCF treatment.

Figure S2. SEM photographs of the ENZ ITO nanolayer.

Figure S3. FTIR spectra of silica glass substrate before and after SCF oxidation.

Figure S4. Visualization of the mechanisms for MD enhancement by SCF processing.

S1. Obtaining the λ_{ENZ} and the values of ε_i

In the VASE measurement, we perform 4 independent measurements on each sample. We use a MATLAB script to perform spline interpolation and seek the very point that crosses the wavelength axis within the precision of 10^{-4} nm. As the final result, we take 2 digits after the decimal point. λ_{ENZ} results obtained this way is quite stable and accurate. The standard deviations of the 4 independent measurements are within 0.16 nm, which is significantly smaller than the observed (and calculated) λ_{ENZ} change. The observed change in ε_r and ε_i (shifted λ_{ENZ} and reduced loss) cannot be simply regarded as a parallel shifting of the complex permittivity, because by influencing the related electrical parameters, the Drude model produce two different curves with slightly different shapes, and these changes are not linear nor proportional. Additionally, the results of λ_{ENZ} shifts for SCF oxidation are quite robust. From four repeated experiments and independent measurements, the success rate of SCF oxidation is > 87.5%. As a method of repairing defects and improving electrical properties, SCF techniques are expected to have a greater effect on heavily-doped defect-rich and lower-quality nanolayers, which is quite useful in experimental conditions.

The absolute values of intrinsic loss ε_i are highly related fabrication techniques, therefore in the main text, we focus more on the margin of improvement. For reference, the ε_i in the ENZ region before and after SCF treatment is shown in Fig. S1.

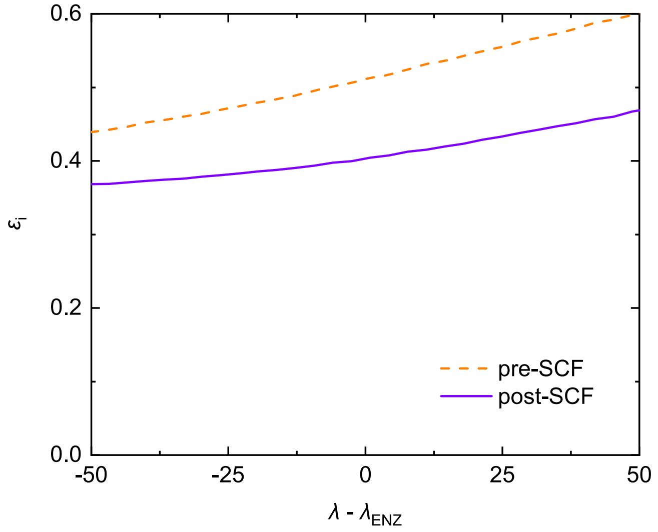

Figure S 1. Intrinsic loss in the ENZ region before and after SCF treatment.

S2. Determination of experimental parameters

CO_2 is easy to enter supercritical state (supercritical point at ∼31.0°C, 1070 psi)[1] where it exhibits a density like a liquid, and viscosity and diffusion coefficient like a gas. Therefore, any parameters above the supercritical point within the supercritical phase are, in theory, usable in the experiment. The reaction conditions of 120°C, 3000 psi, 1 hour are a set of the optimal empirical values that we tried among other combinations. Lower temperature (e.g. 60°C), lower pressure (e.g. 1500 psi), or shorter time (e.g. 30 minute) will lead to a worse result (insufficient reaction), while higher values exhibit insignificant improvement. The parameter set of (120°C, 3000 psi, 1 hour) is determined to be the most suitable for the SCF technique demonstration in ENZ photonic property manipulation. Therefore, in this work we select this set of experimental parameters for demonstration purposes. For other future applications, the optimal parameters for temperature, pressure, and time can vary.

S3. SEM results

SEM photographs are taken at a zoom of 10,000× to observe any damages that could occur which might compromise ENZ ITO's optical properties and performance in applications. The results are shown in Fig. S2 with no observable change.

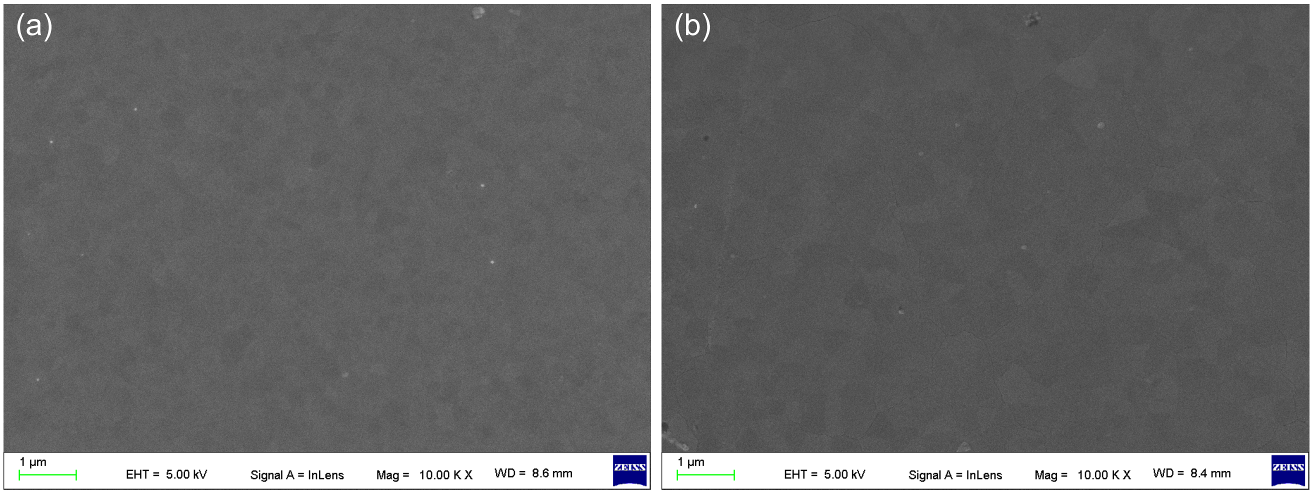

Figure S 2. SEM photographs of the ENZ ITO nanolayer. (**a**) Before and (**b**) After SCF treatment.

S4. FTIR results of substrate

To rule out any possible influences from the silica glass substrate, we measure the FTIR baseline of the substrate before SCF, and re-measure each substrate that goes through different SCF processing with the samples. The results are shown in Fig. S3.

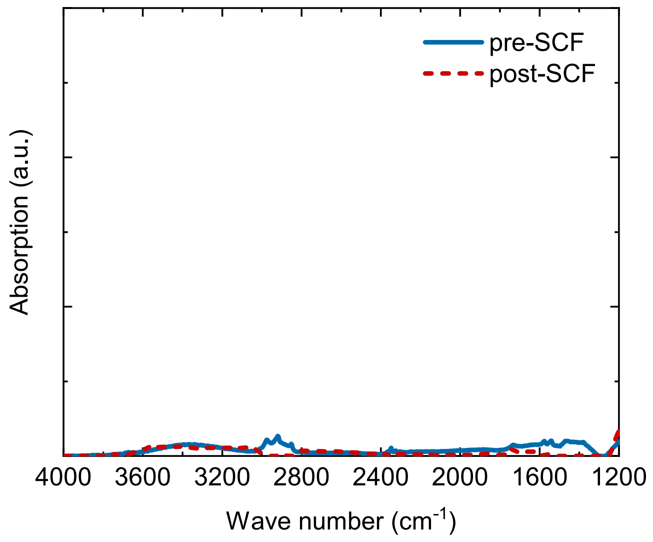

Figure S 3. FTIR spectra of silica glass substrate before and after SCF oxidation.

From Fig. S3, no significant changes can be found in the wavenumber range of interest (3600 cm^{-1} to 3900 cm^{-1}).

S5. Visualize the SCF's enhancement on the MD of nonlinear SA

The following figure is a visualization of the explanation on why SCF processing can enhance the MD of nonlinear SA by repairing defects.

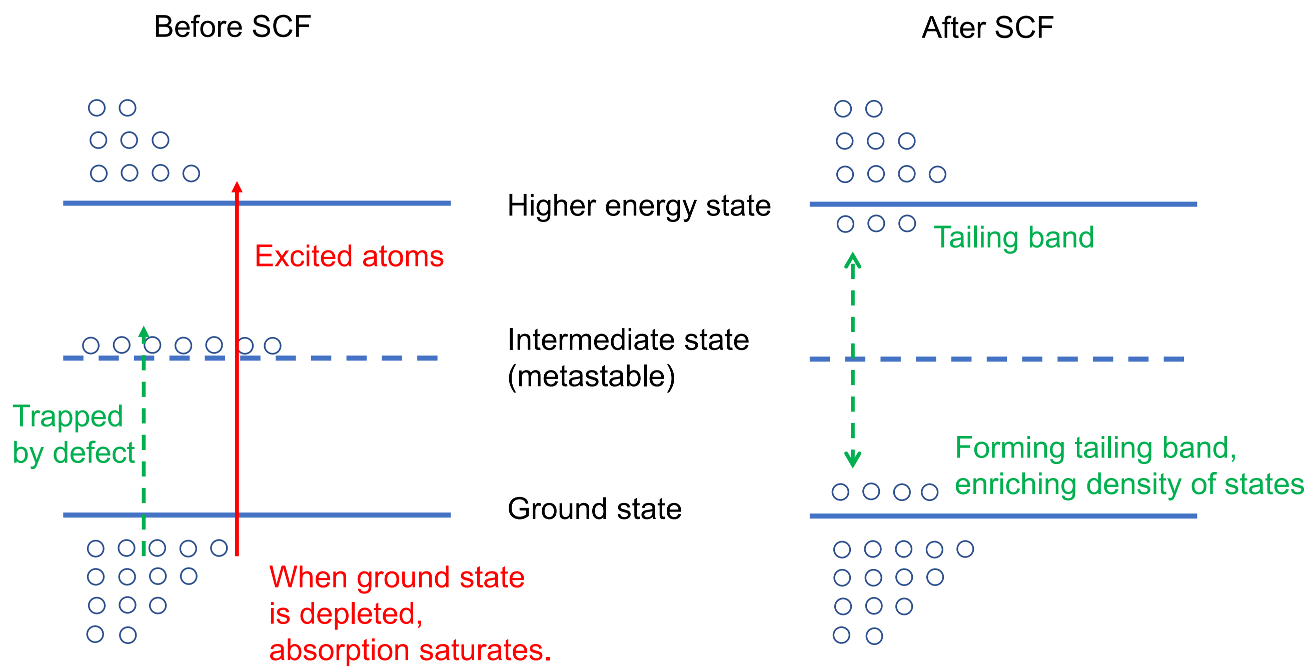

Figure S 4. Visualization of the mechanisms for MD enhancement by SCF processing.

References

1. Span, R. & Wagner, W. A New Equation of State for Carbon Dioxide Covering the Fluid Region from the Triple-Point Temperature to 1100 K at Pressures up to 800 MPa. *J. Phys. Chem. Ref. Data* **25**, 1509–1596, DOI: 10.1063/1.555991 (1996).
